# Deep-Sea Coral Garden Invertebrates and Their Associated Fungi Are Genetic Resources for Chronic Disease Drug Discovery

**DOI:** 10.3390/md19070390

**Published:** 2021-07-13

**Authors:** Pietro Marchese, Ryan Young, Enda O’Connell, Sam Afoullouss, Bill J. Baker, A. Louise Allcock, Frank Barry, J. Mary Murphy

**Affiliations:** 1 Regenerative Medicine Institute, School of Medicine, National University of Ireland Galway, H91W2TY Galway, Ireland; frank.barry@nuigalway.ie; 2 Department of Chemistry, University of South Florida, Tampa, FL 33620, USA; bjbaker@usf.edu; 3 Martin Ryan Institute, School of Natural Sciences, National University of Ireland Galway, University Road, H91TK33 Galway, Ireland; ryan.young@nuigalway.ie (R.Y.); S.AFOULLOUSS1@nuigalway.ie (S.A.); louise.allcock@nuigalway.ie (A.L.A.); 4 Genomics and Screening Core, National University of Ireland Galway, H91W2TY Galway, Ireland; enda.oconnell@nuigalway.ie

**Keywords:** High-throughput screening, hMSC, anti-inflammatory, regenerative medicine, marine fungi, natural products, osteoarthritis, osteoporosis

## Abstract

Chronic diseases characterized by bone and cartilage loss are associated with a reduced ability of progenitor cells to regenerate new tissues in an inflammatory environment. A promising strategy to treat such diseases is based on tissue repair mediated by human mesenchymal stem cells (hMSCs), but therapeutic outcomes are hindered by the absence of small molecules to efficiently modulate cell behaviour. Here, we applied a high-throughput drug screening technology to bioprospect a large library of extracts from Irish deep-sea organisms to induce hMSC differentiation toward musculoskeletal lineages and reduce inflammation of activated macrophages. The library included extracts from deep-sea corals, sponges and filamentous fungi representing a novel source of compounds for the targeted bioactivity. A validated hit rate of 3.4% was recorded from the invertebrate library, with cold water sea pens (octocoral order Pennatulacea), such as *Kophobelemnon* sp. and *Anthoptilum* sp., showing the most promising results in influencing stem cell differentiation toward osteogenic and chondrogenic lineages. Extracts obtained from deep-sea fungi showed no effects on stem cell differentiation, but a 6.8% hit rate in reducing the inflammation of activated macrophages. Our results demonstrate the potential of deep-sea organisms to synthetize pro-differentiation and immunomodulatory compounds that may represent potential drug development candidates to treat chronic musculoskeletal diseases.

## 1. Introduction

Mesenchymal stem/stromal cells (MSCs) are progenitor cells originally identified in bone marrow as a population of undifferentiated cells with a fibroblastic-like phenotype that can differentiate to osteogenic, chondrogenic and adipose lineages in vitro [1]. Data has also shown the capacity of the cells to form bone, cartilage and fat cells in vivo [2,3]. Production of new tissue in vitro and in vivo can be influenced by several factors, such as progenitor cells themselves, the microenvironment and stimulation by growth factors and/or other molecules. The origin of progenitor cells influences the tissue that can be obtained: molecular stimulation of hMSCs leads to a cartilage template later calcified to form endochondral bone, while culture and differentiation of cartilage progenitors form chondrocytes capable to form articular cartilage [4]. Tissue engineers have experimented various biomaterials, such as collagen or marine-derived calcium phosphate matrices, as scaffold structures to promote cell homing and differentiation [5]. Molecular stimulation to drive cell fate can be mediated by a mix of growth factors and synthetic small molecules. The osteogenic commitment of hMSCs in vitro is enabled by peptides from the Bone Morphogenetic Protein (BMP) superfamily and small molecules, such as dexamethasone, β-glycerophosphate and ascorbic acid [6,7,8]. On the other hand, chondrogenic differentiation is mediated by peptides of the Tumor Growth Factor β (TGFβs) superfamily, BMPs and/or small molecules, such as dexamethasone [1,9,10]. Human MSCs treated with pro-osteogenic compounds synthetize intramembranous bone with an extracellular matrix (ECM) characterized by the presence of mineralized calcium, while cells treated with pro-chondrogenic compounds synthetize a proteoglycan ECM characterized by an increased content of sulphated glycosaminoglycans (*s-*GAG). Upon chondrogenic in vitro stimulation, hMSCs undergo differentiation leading to the production of a cartilage template as an intermediate for ultimate production of endochondral bone. Despite observed strong bioactivity ex vivo, bone regeneration induced by BMPs tested in randomized clinical trials showed discordant results [11,12]. For example, TGFβ treatment in vivo induced fibrosis and osteophyte formation [13], and dexamethasone showed significant side effects by preventing cell proliferation and ultimately causing osteoporosis after prolonged treatment [14]. The discovery of new compounds with bioactivity toward hMSCs to regulate their differentiation is therefore required to efficiently induce tissue regeneration in vivo without undesired side effects. Local inflammation also characterizes degenerative diseases: in osteoarthritis, the overexpression of pro-inflammatory cytokines induces cartilage degeneration and undesired calcification with osteophyte formation [15,16], while in atherosclerotic blood vessels they generate a pro-osteogenic environment leading to vessel calcification and plaque development [17,18]. Main pro-inflammatory cytokines implicated in these processes are Interleukin-1 beta (IL1β) and Tumor Necrosis Factor alpha (TNFα), and their targeted inhibition has shown amelioration of osteoarthritis symptoms in diseased animal models [16,19]. The development of new anti-inflammatory drugs specifically designed for degenerative diseases would ideally target the inhibition of IL1β and TNFα.

Deep-sea organisms have evolved under extreme environmental conditions that have influenced the development of different biochemical functions compared to shallow- water organisms [20,21]. Despite the much wider extension of deep compared to shallow marine habitats, approximately 2% of over 30,000 marine compounds isolated up to 2014 were derived from deep-sea organisms [22,23], and approximately 75% of these molecules showed notable bioactivity. This trend improved in following years due to an increased technological capacity for deep-sea exploration and a very high bioactivity hit-rate of deep-sea derived molecules in drug screenings. Schupp and co-workers [24] reported 74% of extracts from deep-sea sponges and gorgonians to be bioactive in anti-cancer assays, while Dumdei and colleagues [25] reported a two-fold higher rate of anti-cancer activity in deep-sea extracts compared to a collection of shallow-water extracts. A computational analysis to investigate the drug comparability of recently discovered deep-sea natural products was performed by Pilkington [26], whose work highlighted 40% of the selected compounds to be drug-like. Corals and sponges have been extensively studied as scaffold material, but rarely as a source of compounds influencing stem cell differentiation [27]. Certain species are able to synthetize protein and a glycosaminoglycan-based extracellular matrix that can calcify to create solid structures [28] through a process similar to animal bone development [29]. Moreover, analogues of human BMPs were isolated from shallow -water corals [30]. Fungi as producers of statins [31] are amongst the first organisms highlighted for their ability to generate small molecules capable of promoting osteogenesis, and frequently reported for the production of anti-inflammatory compounds [32].

Recently, we showed that itaconate derivatives isolated from a marine strain of *Penicillium antarcticum* were able to inhibit osteogenic differentiation of bone marrow derived MSCs; this was achieved by implementing a High-Throughput Screening (HTS) platform developed to detect hits for regenerative medicine [33]. To expand this drug discovery program, in the present study we bioprospected a large library of extracts obtained from deep-sea corals, sponges and fungi, aiming at the identification of organisms able to synthetize compounds bioactive toward hMSCs and/or able to inhibit macrophage pro-inflammatory activity. Screening results were used to direct targeted chemical studies to facilitate the isolation and structure elucidation of drug candidates from promising deep-sea species for the treatment of chronic diseases.

## 2. Results

### 2.1. Invertebrate Extract Bioactivity toward hMSCs

**Figure 1:**
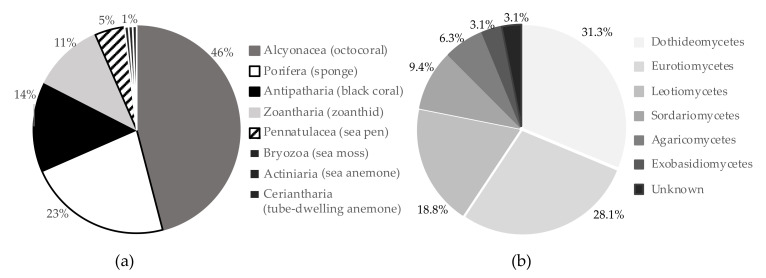
Proportion of each group of organisms in the extract libraries. (**a**) deep-sea invertebrate extract library (**b**) deep-sea fungi extract library.

**Figure 2:**
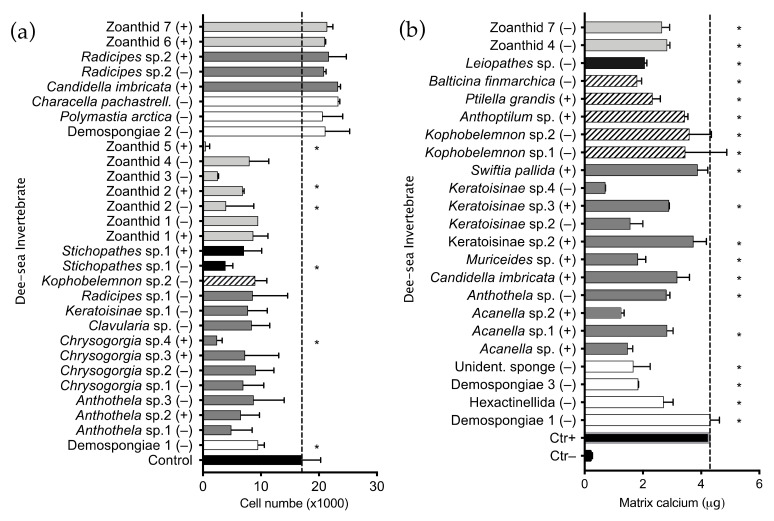
Deep-sea invertebrate extract bioactivity toward hMSCs. Cells were treated with 150 µg/mL extracts diluted in basal medium (IOM) lacking the pro-osteogenic drug dexamethasone. Positive control cells (Ctr+) were treated with IOM supplemented with 200 nM dexamethasone (OM) and negative control cells (Ctr−) were treated with IOM alone. Plates were incubated for 10 days and ECM mineralized calcium was analyzed as a marker of osteogenic differentiation. Cell proliferation was quantified after treatment on sister plates by counting nuclei of living cells in each well. (**a**) Pro-osteogenic hit extracts selected from the HTS using the screening threshold (Ctr+ average + 3*standard deviation) were statistically compared to the negative control (Ctr−, cells cultured without the pro-osteogenic drug dexamethasone) to confirm significant bioactivity. (**b**) Hit extracts were selected from the HTS based on the ability to induce variation in cell number above or below the screening threshold. Anti-proliferative extracts (values below the dashed line) were statistically compared to the initial number of seeded cells (10^4^), while proliferative hits (values higher than Control, last eight in the histogram) were compared to the untreated control average. The screening was performed on experimental duplicates. Data are presented as the mean ± SD, * indicates *p* ≤ 0.05 calculated using ANOVA one-way with Bonferroni post-test. (+) indicates the polar extract fraction obtained with methanol, while (–) indicates the non-polar extract fraction obtained with dichloromethane.

**Figure 3:**
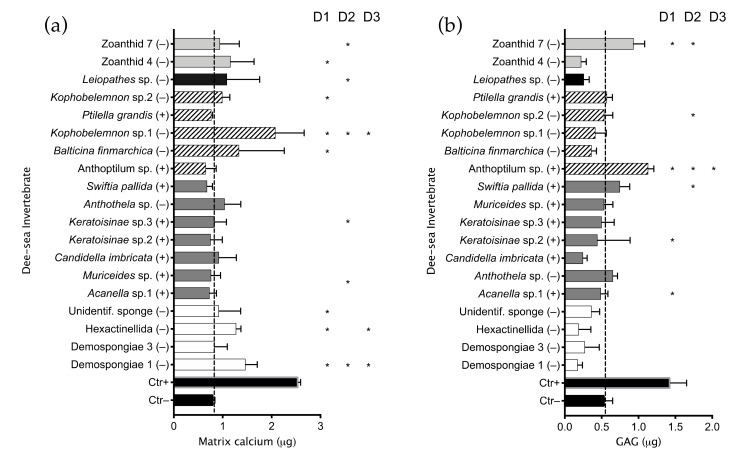
Deep-sea invertebrate extracts bioactivity validation. (**a**) Nineteen pro-osteogenic extracts selected from the HTS were tested in triplicate on three hMSC donors (D1, D2, D3) with the same procedure used in the automated screening to validate extracts bioactivity for hMSC osteogenic differentiation. (**b**) Cells chondrogenic differentiation was tested by quantifying the *s-*GAG production by three hMSC donors after treatment with selected pro-osteogenic hits. Crude extracts were diluted in ICM medium lacking the pro-chondrogenic factor TGF-β3 and compared to negative control cells (Ctr−) treated with ICM, or positive control cells (Ctr+) treated with ICM and 10 ng/mL TGF-β3. The screening was performed in triplicate. Results are presented as the mean ± SD, * indicates *p* ≤ 0.05 calculated using ANOVA one-way with Bonferroni post-test. (+) indicates the polar extract fraction obtained with methanol, while (−) indicates the non-polar extract fraction obtained with dichloromethane.

A library of four 96-well plates containing 320 polar and non-polar extracts was tested using our HTS platform to find deep-sea invertebrates synthetizing compounds influencing growth and differentiation of human MSCs. The chemical library was composed of extracts obtained from 179 invertebrate taxa: 111 belonging to the phylum Cnidaria, 66 Porifera and one Bryozoa (Figure 1a). Extracts obtained from alcyonaceans (octocorals, order Alcyonacea) represented 46% of the library (147 extracts, 68 different taxa); black coral (hexacorals, order Antipatharia) extracts represented 14% (45 extracts, 22 different taxa); zoanthids (hexacorals order Zoantharia) were 11% (35 extracts, 12 taxa); sea pens (octocorals, order Pennatulacea) 5% (15 extracts, 8 taxa). Sea mosses (Bryozoa), sea anemones (Actiniaria) and tube-dwelling anemones (Ceriantharia) represented 1% of the library each (2 extracts, 1 taxon per animal), sponges (phylum Porifera) were 23% of the library (72 extracts, 66 taxa). The library screening highlighted 21 extracts that negatively influenced hMSC proliferation and eight extracts promoting proliferation (Supplementary Figure S1b). Ten anti-proliferative extracts were obtained from soft coral, seven from zoanthid, two from black coral, one from a sponge and one from a sea pen. Extracts influencing proliferation of untreated cells were obtained from three soft coral, three sponge and two zoanthid extracts (Figure 2a). Cytotoxic extracts were also detected in this screening; three extracts obtained from zoanthid, two from soft coral, and one from black coral (Figure 2a). The bioactivity and polarity of extracts is summarized in Table 1. High-throughput screening for pro-osteogenic factors showed 23 extracts inducing cell calcium mineralization higher than the screening selection threshold. Eleven pro-osteogenic extracts were obtained from soft coral, five from sea pen, four from sponge, two from zoanthid and one from black coral (Figure 2b). Comparison of results from the two assays and classical statistical analysis (ANOVA one-way) was used to select positive pro-osteogenic and non-toxic hits for further testing. Out of 23 hits selected from the HTS using this threshold, 19 extracts significantly improved calcium mineralization in comparison to the untreated control and showed no significant cytotoxicity. These 19 hits were selected for further testing using three different hMSC donors. The ability of extracts to influence cell differentiation leading to endochondral bone formation was evaluated by measuring extracellular matrix (ECM) calcium mineralization and *s-*glycosaminoglycans (s-GAG) content. Nineteen pro-osteogenic and non-toxic extract hits were re-screened; 11 were shown to retain pro-osteogenic bioactivity and significantly induced calcium mineralization in at least one MSC donor (Figure 3a). Extracts obtained from an unidentified demosponge and from the sea pen *Kophobelemnon* sp.1 induced differentiation in all three donors tested, while a hexactinellid sponge extract induced differentiation in two out of three donors. Eight extracts induced differentiation in only one donor tested: the soft corals *Muriceides* sp. and Keratoisinae sp.3, the sea pen *Balticina finmarchica*, the black coral *Kophobelemnon* sp.2 and *Leiopathes* sp. and two unidentified zoanthids Zoanthid 4 and 7. Evaluation of the extracts’ influence on hMSC chondrogenic differentiation showed six extracts inducing ECM expression of *s-*glycosaminoglycans significantly higher than the untreated control (Figure 3b). Extracts obtained from the sea pen *Anthoptilum* sp. influenced chondrogenic differentiation of all three donors tested while Zoanthid 4 induced differentiation in two out of three donors. Four extracts induced differentiation in only one donor tested: the soft corals *Acanella* sp.1, Keratoisinae sp.2, *Swiftia pallida* and the sea pen *Kophobelemnon* sp.2.

### 2.2. Fungal Extract Bioactivity toward hMSCs

**Figure 4:**
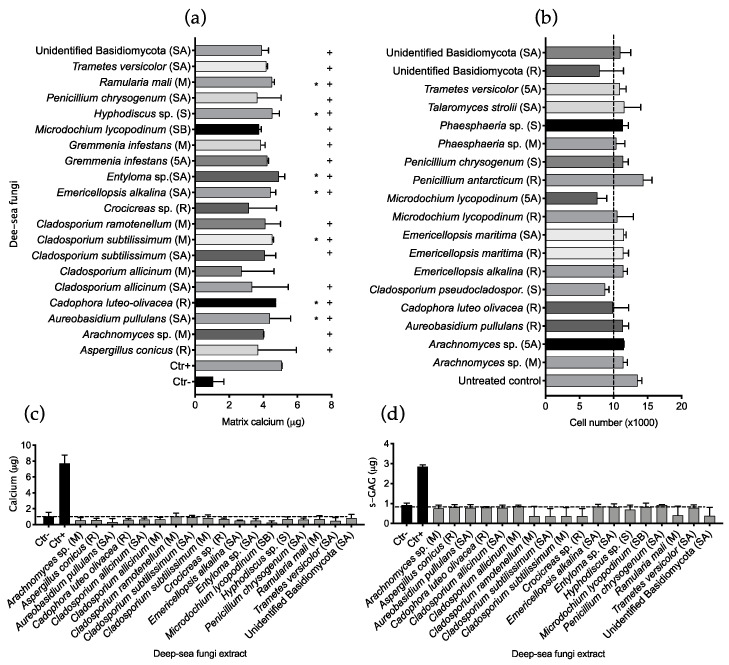
Deep-sea fungal extracts bioactivity toward hMSCs. (**a**) Cell number retrieved after treatment with deep-sea fungi extract: ten thousand cells were seeded in 96-well plates, exposed to 125 µg/mL extracts diluted in IOM medium or IOM only (Control) and incubated for 10 days. After incubation, number of live cells in each well was counted using the Operetta system after nuclear staining. Hit extracts were selected from the HTS as inducing variation in cell number above or below the screening threshold (Ctr+ average + 3*standard deviation). Bioactivity of selected hits was statistically compared to the untreated Control. Cytotoxicity was evaluated by comparing the number of cells recorded to the initial number of seeded cells (dashed line). (**b**) hMSCs osteogenic differentiation after treatment with deep-sea fungi extracts: cells were treated as described above, and an additional control (Ctr+) represented the cell treatment with OM medium containing IOM with 100 nM of dexamethasone. After incubation, cell medium was discarded and ECM mineralized calcium was quantified as marker of osteogenic differentiation. Pro-osteogenic hit extracts selected from the HTS using the screening threshold (Ctr+ average + 3*standard deviation) and compared to the negative control (Ctr−) to confirm significant bioactivity. (+) indicates the extracts selected for further screening. (**c**) Eighteen pro-osteogenic extracts selected from the HTS were tested in triplicate on three hMSCs donors to validate the extracts bioactivity. (**d**) Cells chondrogenic differentiation was tested by quantifying the *s-*GAG production by three hMSC donors after treatment with selected pro-osteogenic hits. Crude extracts were diluted in ICM medium lacking TGF-β3 and compared to negative control cells (Ctr−) treated with ICM, or positive control cells (Ctr+) treated with ICM and 10 ng/mL TGF-β3. For (**a**,**b**), the screening was performed on experimental duplicates, c and d were performed in triplicate. Results are presented as the mean ± SD, * indicates *p* ≤ 0.05 calculated using ANOVA one-way with Bonferroni post-test. In brackets is reported the growing medium from which the bioactive extract was obtained: R = rice; SA = rice + suberohydroxamic acid; SB = rice + sodium butyrate; 5A = rice + 5-azacytidine; M = Malt extract agar; S = Soy mannitol agar.

A library of two 96-well plates containing 160 fungal extracts was tested using the HTS platform to detect compounds able to influence hMSC differentiation and proliferation. The library was composed of extracts obtained by 28 Ascomycota species and four Basidiomycota species: ten dothideomycetes, nine eurotiomycetes, six leotiomycetes, three sordariomycetes, two agaricomycetes, one exobasidiomycete and one unknown species (Figure 1b). Thirteen fungi were isolated from deep-sea animal samples and 21 from deep-sea sediments. Seventeen species showed anti-proliferative bioactivity by inducing a cell number lower than the screening threshold after treatment with fungal extracts. Data points outside the threshold were analysed using ANOVA one-way in comparison with the number of initially seeded cells (10^4^) and showed no significant difference in cell number, suggesting an anti-proliferative action rather than cytotoxicity. The high-throughput (HT) osteogenic assay highlighted 22 extracts inducing ECM calcium mineralization above the threshold set for hit detection (Supplementary Figure S2), seven of which showed significance when analyzed in comparison to the untreated control using ANOVA-one way (Figure 4a). 

Eighteen hits from the HTS not showing cytotoxicity (Figure 4b) were selected and re-tested manually on three hMSCs donors to validate retention of bioactivity. The re-screening was performed in triplicate and the potential to influence differentiation of hMSCs was evaluated by quantifying ECM calcium mineralization and *s*-GAG production in two separate experiments. Results from this screening showed no extracts retaining significant bioactivity toward hMSCs to induce osteogenic (Figure 4c) or chondrogenic (Figure 4d) differentiation.

### 2.3. Anti-Inflammatory Bioactivity 

**Figure 5:**
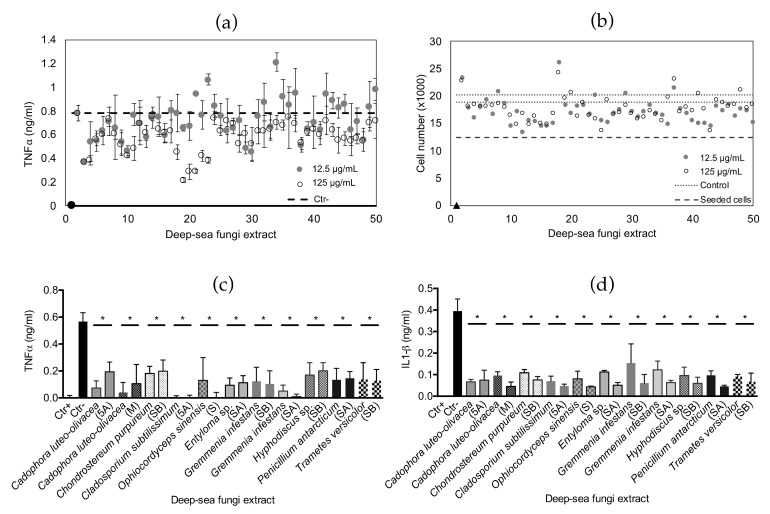
Deep-sea fungal extract anti-inflammatory bioactivity. (**a**) Anti-inflammatory bioactivity of 49 hits selected from the initial HTS: extracts were re-tested using the same procedure, in triplicate. Twelve thousand cells were stimulated with 100 ng/mL LPS, seeded in 384-well plates and exposed to fungal extracts dissolved in growth medium at concentrations of 125 µg/mL and 12.5 µg/mL. Negative control cells (Ctr−, dashed line) were only exposed to LPS while negative control cells (Ctr−, black dot and continuous grey line) were seeded in growth medium only. After 6 h incubation, the pro-inflammatory cytokine TNFα was quantified. (**b**) THP1 cell viability after treatment with 49 bioactive hits: extract re-screening was measured by aseptically adding 10% alamarBlue to the cell conditioned medium (after 6 h incubation with the inflammatory signal) and incubating the plates for an additional 8 h. Cell number was calculated by comparing experimental results to a standard curve prepared with cells at increasing concentrations initially seeded in the screening plate. Averages of positive (Ctr+) and negative (Ctr−) control cell viability are shown as dotted lines, while the level of the initial cells seeded (12 × 10^3^) is indicated with a dashed line. A cytotoxic control of cells treated with 10% DMSO was also included (black triangle on bottom left). Eleven anti-inflammatory fungal extract hits were validated on activated macrophages using the same procedure by testing cell release of pro-inflammatory cytokines TNFα (**c**) and IL1β (**d**). In both cases extracts were tested at 125 µg/mL and 12.5 µg/mL and individually compared to the cytokine level released by LPS treated cells (Ctr−) using ANOVA one-way. In both cases results are presented as the mean ± SD; * indicates *p* ≤ 0.05 calculated using ANOVA one-way with Bonferroni post-test. The growth medium from which the bioactive extract was obtained is shown in brackets: R= rice; SA= rice + suberohydroxamic acid; SB= rice + sodium butyrate; 5A= rice + 5-azacytidine; M= Malt extract agar; S= Soy mannitol agar.

The fungal library was also tested for extracts containing immunomodulatory compounds. The anti-inflammatory HT assay was successfully scaled up from a 96-well to a 384-well plate format: an optimal Z’ factor (>0.7) was obtained using the screening conditions selected (Supplementary Materials, Figure S3). After incubation, the pro-inflammatory cytokine TNFα was quantified in the conditioned medium as a marker of cell inflammation (Supplementary Figure S4). Hit extracts were selected when inducing TNFα levels in the conditioned medium lower than cells treated with LPS and no other compound (negative control average). Forty-nine hit extracts were selected from the HTS and further tested at two concentrations (125 and 12.5 μg/mL) on THP1 cells using the same procedure but performing three experimental replicates for each extract and concentration (Figure 5a). Thirty-two hits were confirmed anti-inflammatory when tested at 125 μg/mL, while only 15 extracts showed a significant decrease of TNFα when tested at 12.5 μg/mL. Fourteen extracts induced significant decreases of the pro-inflammatory cytokine at both concentrations tested; three of these were not available for further screening, while eleven extracts were further validated. Cell viability after treatment with the selected 49 hits (Figure 5b) showed no significant variation compared to the untreated controls, highlighting an absence of cytotoxicity for the macrophage cell line. 

The eleven selected extracts were manually tested following the same procedure, while inflammation was evaluated by quantification of the pro-inflammatory cytokines TNFα and IL1β. Extracts re-tested were obtained by *Cadophora luteo-olivacea* growing on an MEA medium and *C. luteo-olivacea* treated with 5-azacytidine; *Chondrostereum purpureum* treated with sodium butyrate; *Cladosporium subtilissimum* treated with 5-azacytidine; *Gremmenia infestans* treated with suberohydroxamic acid and sodium butyrate; *Hyphodiscus* sp. treated with sodium butyrate; *Penicillium antarcticum* treated with suberohydroxamic acid; *Trametes versicolor* treated with sodium butyrate; *Ophiocordyceps sinensis* growing on SMA and *Entyloma* sp. treated with suberohydroxamic acid. All eleven extracts were shown to significantly reduce the inflammation state of the THP1 macrophages at both concentrations tested, inducing a reduction of the pro-inflammatory cytokines TNFα (Figure 5c) and IL1β (Figure 5d) released by LPS -activated macrophages. 

## 3. Discussion

Deep sea is the widest natural environment existing on Earth and hosts the vast majority of marine habitats worldwide. Despite its size, only 5% of the deep sea has been explored using remote instruments, and only a fraction of its floor has been sampled and studied in detail [34]. Therefore, this habitat still represents a huge untapped area for ecological and biotechnological discoveries. Unlike terrestrial or coastal natural habitats, deep-sea research is currently facilitated by limited regulations to protect genetic resources from areas beyond national jurisdictions, allowing its access for exploratory activity [35]. Logistic and technological limits to deep-sea exploration are also being overcome by the increasing availability of Remotely Operated Vehicles to academic scientists through government-owned research vessels or in collaboration with marine-based companies [36]. Coral gardens are rich and diverse deep-sea habitats populated by cold-water corals and sponges forming complex communities on rock formations, canyons or the deep-sea bed [37]. Filamentous fungi are found to live in association with benthic invertebrates as well as in the surrounding sediments [38], with ecological roles still to be determined. The chemical capabilities of microbes and benthic animals living in the deep sea has not been thoroughly explored, representing a knowledge gap in the marine natural products drug discovery field. 

In this study we investigated the potential of invertebrates and filamentous fungi from a deep-sea coral garden to synthetize bioactive compounds to treat chronic diseases associated with bone and cartilage degeneration. We implemented a previously developed HTS platform for drug discovery in regenerative medicine [33] to detect bioactivity and generate data for targeted chemical investigation. A library of crude extracts from deep-sea invertebrates was tested on hMSCs to evaluate their ability to induce differentiation toward osteogenic and chondrogenic lineages. A second library of deep-sea fungal extracts was tested on hMSCs and on activated macrophages to evaluate the production of immunomodulatory compounds. Automated HTS of the invertebrate library showed 23 hits, representing 7.2% of all extracts tested. Eleven of these were confirmed bioactive on at least one hMSC donor, representing a 3.4% bioactivity hit rate. The most promising organisms producing pro-osteogenic compounds, i.e., those inducing significant differentiation in all three donors tested, were obtained from a specimen of an unidentified demosponge and from the sea pen *Kophobelemnon* sp.1 (octocoral order Pennatulacea), while an hexactinellid sponge extract induced differentiation in two out of three donors and eight other extracts from soft coral, black coral, zoanthid and sea pen were confirmed osteogenic for only one hMSC donor. The hMSC chondrogenic differentiation was also influenced by six extracts that induced significant increases of the cartilage-associated extracellular matrix component *s-*GAG [1]. The most promising bioactive extract was obtained from the sea pen *Anthoptilum* sp., which induced differentiation in all three donors tested. The second most reliable bioactive extract was obtained from an unidentified zoanthid (Zoanthid 7), which induced differentiation in two out of three donors, while two soft corals and one black coral induced differentiation in only one donor. Comparing the bioactivity obtained by different animal groups, the combined (osteogenic and chondrogenic differentiation) hit rate showed sea pen extracts as having the best performance with a 27% hit rate, followed by zoanthids (6%), sponges (4%), soft corals (3%), and black corals (2%). Eleven out of nineteen hits were represented by non-polar extracts and eight from polar extracts. Considering the performance on hit-rate recorded from sea pens, the strong pro-osteogenic bioactivity by *Kophobelemnon* sp.1 and strong pro-chondrogenic bioactivity by *Anthoptilum* sp., this group of deep-sea benthic invertebrates is the most promising for the discovery of drug candidates to treat chronic diseases of bone and cartilage.

The growth and survival of hMSCs in culture was also influenced by deep-sea invertebrate compounds. Twenty-one extracts inhibited cell proliferation while six showed significant cytotoxicity when compared to the initial number of seeded cells. In terms of hit rate, zoanthids showed the highest score with 25.7% of extracts in the library being antiproliferative or cytotoxic, followed by soft coral (8.8%), sea pen (6.6%), sponge (5.5%) and black coral (4.4%) extracts that only showed anti-proliferative activity in one case. Extracts obtained by zoanthids also showed the highest cytotoxicity, causing up to 94% of cell death. According to the screening threshold, eight extracts showed proliferative influence on hMSCs: three from sponge extracts, three from soft coral and two from zoanthid, but this result was not supported by significant statistical power. Overall, deep-sea zoanthids showed the highest performance in producing compounds influencing the cell proliferation cycle, either by killing them, stopping their reproduction, or influencing a faster division compared to untreated control cells. The deep-sea fungal extracts library was tested for differentiation induction of hMSCs, using the same procedure performed for the invertebrate library. Twenty extracts out of 160 were shown to induce osteogenic differentiation in hMSCs, but out of 18 extracts re-tested, none were demonstrated to be a reliable positive hit. The high number of false positives in complex cell-based screenings underpins the value of multiple testing to validate hits before prioritization for detailed chemical investigations or scale-up. The fungal library was also tested for the detection of immunomodulatory compounds, implementing a procedure successfully scaled-up to a 384-well plate format for both cell screening and ELISA. Our method, supported by a positive Z’ factor, allowed simultaneous testing of multiple conditions without affecting a time-sensitive procedure for both the cell screening and immunoassay. Obtained results identified 49 extracts influencing a decreased release of pro-inflammatory cytokines, 32 of which were further validated and showed no cytotoxicity on THP1 cells. Eleven immunomodulatory extracts were selected according to their biomass availability and for showing bioactivity at lower concentrations. All extracts were shown to significantly reduce the production of the pro-inflammatory cytokines TNFα and IL1β, representing valid candidates for anti-inflammatory drug discovery. Such bioactive compounds would be particularly useful to treat chronic musculoskeletal conditions where these cytokines are responsible for triggering and maintaining disease aetiology in conditions like osteoarthritis [39].

**Figure 6:**
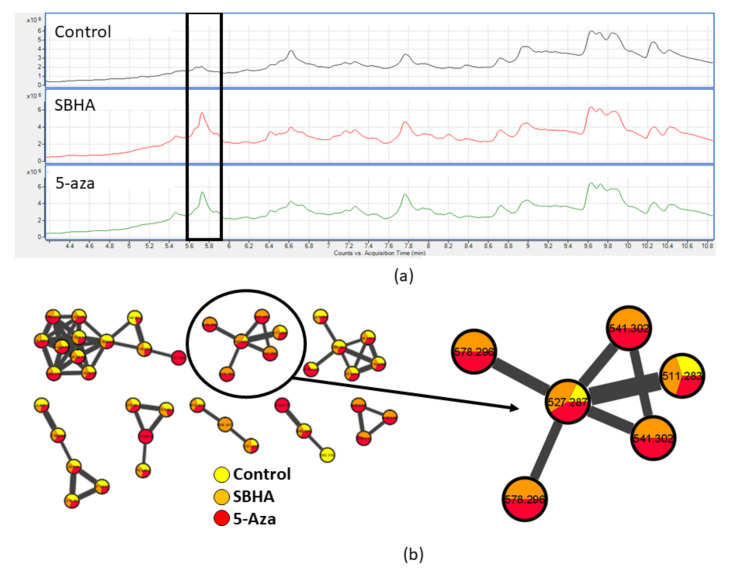
LC-MS Metabolomic Analysis of the extracts of *Gremmenia infestans***.** (**a**) LC-MS traces of extracts *Gremmenia infestans* grown in the absence of inhibitors (*above*), in the presence of SBHA (*middle*) or 5-azacytadine (*bottom*). The outlined box around the peak at 5.73 min denotes the upregulation of the fungal metabolite corresponding to a *m*/*z* = 527.2814. (**b**) Image of the MSMS molecular networking, of the extracts of *Gremmenia infestans* grown under the three conditions. Each node is labeled with the parent mass ion, with a pie chart denoting the relative abundance. The width edges connecting the nodes correspond to a cosine score denoting the fragment similarity between each node. The cluster corresponding to the upregulated in metabolite in (**a**) is expanded.

The implementation of the OSMAC approach to grow fungi for chemical extraction proved to be an optimal strategy to stimulate the production of bioactive compounds, as previously described [40], and highlighted once again the necessity to apply a range of growth conditions to comprehensively test fungal metabolic potential for drug discovery. Nine out of eleven bioactive fungal extracts were obtained by fungi treated with epigenetic modifiers: four were treated with the HDAC inhibitor suberohydroxamic acid, three with sodium butyrate and two with the DNMT inhibitor 5-azacytidine. In most cases (6/11) a single epigenetic modifier was effective in inducing the production of bioactive compounds. Both HDAC inhibitors induced the production of bioactive extracts in *Gremmenia infestans*, while *Cadophora luteo-olivacea* synthetized bioactive compounds in both the control medium and under stimulation with the DNMT inhibitor used. Metabolomic analysis of *Gremmenia infestans* showed a 20X upregulation of a metabolite (*m*/*z* = 527.2814, retention time 5.73 min) when treated with the HDAC inhibitors (Figure 6a). MSMS molecular networking [41] of these three fungal extracts yielded eight major clusters (Figure 6b), two of which had metabolites produced in the presence of the epigenetic modifiers. This example supports our rationale for screening a single species of fungus under several growth conditions. This methodology also allowed us to tentatively identify the metabolites responsible for the biological activity observed in our assays. The entomopathogenic fungus *Ophiocordyceps Sinensis* showed synthesis of bioactive compounds when not treated with epigenetic modifiers.

## 4. Materials and Methods

### 4.1. Deep-Sea Organism Collection

Samples of cold-water corals and sponges were collected from deep-sea gardens located in the Whittard Canyon during expedition CE17008 of *RV Celtic Explorer*, while sediments were collected along the deep margin of the west European Continental shelf on the Porcupine Banks during expedition CE18012. In each expedition, samples were collected with the Remotely Operated Vehicle (ROV) Holland I and stored at −80 °C before chemical extraction. Samples for microbial isolation were surface sterilized and stored in glycerol as cryoprotectant (see [38] for more details on samples preparation and storage). Deep-sea fungi in pure culture are cryopreserved at the National University of Ireland Galway, Ireland.

### 4.2. Deep-Sea Benthic Invertebrate Metabolite Extraction

Animal samples were photographed on board, catalogued and immediately frozen at −80 °C. Samples were lyophilized prior to extraction. Coral samples were exhaustively extracted in dichloromethane using a Soxhlet. The macerated corals were then allowed to air dry to remove any remaining DCM, and subsequently extracted in methanol under sonication for 30 min. Both extracts were filtered and concentrated under reduced pressure, yielding a lipophilic and polar extract for each coral. Dry sponges were macerated in a mixture of dichloromethane and methanol (1:1) and sonicated. The solvent was filtered and concentrated yielding an apolar extract. The remaining sponge tissue was allowed to dry before an additional extraction by sonication in aqueous methanol (1:1). The filtrate was then dried under vacuum yielding a polar extract. All extracts were re-solubilised in dimethyl sulfoxide (DMSO) at a concentration of 30 mg/mL, added to 4 96-well plates and stored at −20 °C before screening. 

### 4.3. Deep-Sea Fungal Culture and Metabolite Extraction

A selection of 32 fungal strains isolated from deep-sea sediment or invertebrates (see Marchese et al., 2021 for information on fungal isolation and identification) were cultivated following an OSMAC approach [42]. To induce the activation of variable biosynthetic gene clusters, each fungal strain was treated with epigenetic modifiers. For each isolate, two 0.5 cm^2^ pieces of fungal biomass on agar were inoculated into 1.25 mL of either: (1) untreated Malt Extract Broth (MEB); (2) 100 μM sodium butyrate in MEB; (3) 100 μM 5-azacytadine in MEB; or (4) 100 μM suberohydroxamic acid in MEB, and incubated overnight at 24 °C. After incubation, each aliquot containing medium and biomass was poured over 3 g of autoclaved brown rice in a 20 mL scintillation vial. Cultures were incubated at 24 °C for 28 days. Each strain was also inoculated on solid media: Malt Extract Agar, Soy Mannitol Agar, Potato Dextrose Agar and Sabouraud dextrose Agar as backup biomass for species capable of growing on rice. Following incubation, fungal biomass along with growth medium were sprayed with approximately 500 μL MeOH and 10 mL ethyl-acetate (EtOAc) added. Cultures were kept on shaker for 24 h with the extract, then filtered, dried, and re-dissolved to a concentration of 25 mg/mL in DMSO. In total, 180 crude extracts were transferred into 2 96-well plates for bioassays and chemical analysis and stored at −20 °C before screening. 

### 4.4. Culture of Human Mesenchymal Stem Cells and THP1 Macrophages

Primary hMSCs were isolated from bone marrow obtained from healthy donors, Galway University Hospital, Galway, Ireland. All procedures followed were in accordance with ethical standards of the responsible local committees for human experimentation (institutional and national) and the Helsinki Declaration of 1975, as revised in 2000, and informed consent was obtained from all marrow donors included in the study. Isolated hMSCs were expanded in a growth medium containing α-minimal essential medium (α-MEM, Life technologies, Carlsbad, CA, USA), 10% foetal bovine serum (FBS, Sigma, St. Louis, MO, USA) and 1% penicillin/streptomycin (P/S, Life technologies) supplemented with 5 ng/mL fibroblast growth factor-2 (FGF-2, Peprotech, Rocky Hill, NJ, USA) and cultured at 37 °C, 5% CO_2_. The medium was refreshed 3 times weekly and cells were sub-cultured or frozen at 90% confluence in T175 flasks (Sarstedt, Newton, NC, USA). The immortalized human monocytes THP1 were cultured in an RPMI (Sigma) growth medium with 10% FBS and 1% P/S, and incubated at 37 °C, 5% CO_2_. The medium was refreshed 3 times weekly and the cells were sub-cultured or frozen at 90% confluence in T175 flasks (Sarstedt).

### 4.5. Marine Extract Library Screening

A library composed of 2 96-well plates of fungal extracts and 4 96-well plates of benthic invertebrate extracts was tested for the detection of compounds modulating hMSCs osteogenic and chondrogenic differentiation and immunomodulatory bioactivity following previously described procedures [33] with modifications as required.

### 4.6. High-Throughput Osteogenic Assay

Osteogenic differentiation mediated by marine extracts was investigated using HTS technology (Janus Automated Workstation, PerkinElmer). Primary hMSCs cultured for osteogenic screening were used up to Passage 4 (P4–16 to 20 population doublings). Cells were suspended in a basic medium (BM) containing phenol red free Dulbecco’s modified eagle medium low glucose (DMEM-LG, Life Technologies, Carlsbad, CA, USA), 10% FBS and 1% P/S. Ten thousand cells in 100 μL were seeded in optical flat-bottom 96-well plates (Cell Carrier Ultra, PerkinElmer, Waltham, MA, USA) and incubated 6 h at 37 °C, 5% CO_2_ for recovery post-thaw and attachment to the culture surface. To induce osteogenic differentiation, thecells were exposed to an equal volume of Osteogenic Medium (OM) containing BM supplemented with 0.2 μM dexamethasone (Dex, Sigma), 200 μM ascorbic acid 2-phosphate (AA2p, Sigma) and 20 mM β-glycerophosphate (β-gly, Sigma). Marine organism-derived extracts were diluted in an Incomplete Osteogenic Medium (IOM), OM without dexamethasone. Untreated control cells were cultured in an equal volume of BM (200 μL) added of DMSO to a final concentration of 0.5%, equal to the experimental wells. Plates were sealed with Breathe-Easy sealing membrane (Sigma) and incubated at 37 °C, 5% CO_2_ for 10 days. Four replicate plates were prepared per each drug screening in order to obtain 2 replicates to test for differentiation and 2 replicates for cell number quantification. Differentiation was measured as the calcium mineralized in the extracellular matrix: the growth medium was discarded and the cell layer was exposed to 50 μL HCl 0.5 M to solubilize the calcium crystals. After 15 min incubation at room temperature, dissolved calcium was quantified by mixing 10 μL of sample with 190 μL Stanbio colorimetric reagent (Stanbio LiquiColor, Boerne, TX, USA) and absorbance at 405 nm was immediately measured using a Viktor X5 plate reader (PerkinElmer). The cell number was counted through nuclear staining after an addition of 5 μg/mL Hoechst 33342 (Invitrogen, Waltham, MA, USA) to the cells, incubating 15 min in the dark and imaging the plates using the Operetta High Content Imaging System (PerkinElmer). 

Hit selection was performed by setting a screening threshold of three times the standard deviation from the screening control average, as previously described [43,44]. Hits were further investigated when no cytotoxic activity was recorded. The cell number recorded after treatment was used to evaluate effects of the extracts on cell proliferation: the screening threshold was used to detect proliferative extracts (higher than the higher threshold limit) or cytotoxic extracts (lower than the lowest threshold limit). Detected hit re-screening was manually performed using the same procedure and samples tested in triplicate for differentiation induction. Three human MSCs donors were tested to validate retention of bioactivity. 

### 4.7. Chondrogenic Assay

Chondrogenic differentiation mediated by selected pro-osteogenic marine extracts was manually tested using miniaturized assays. Primary hMSCs cultured for chondrogenic screening were used up to Passage 4 (P4–16 to 20 population doublings). Cells were suspended in Incomplete Chondrogenic Medium (ICM) containing DMEM high-glucose phenol red-free (DMEM-HG, Life Technologies) 1% insulin, transferrin and selenium (ITS+) supplement (6.25 μg/mL bovine insulin, 6.25 μg/mL transferrin, 6.25 ng/mL selenous acid, 5.33 μg/mL linoleic acid and 1.25 mg/mL BSA), 50 μg/mL ascorbic acid 2-phosphate, 40 μg/mL l-proline, 100 nM dexamethasone, 1 mM sodium pyruvate and 1% P/S. Fifty thousand cells in 100 μL were seeded in each well of optical flat bottom 96-well plates (Cell Carrier Ultra, PerkinElmer) and incubated overnight to enable cell recovery and aggregate formation. After incubation, 50 μL medium was discarded and cells were exposed to an equal volume of the appropriate media. Positive control cells were treated with CCM (ICM with 20 ng/mL TGFβ-3), while negative control cells were treated with ICM; in both cases cells were added of DMSO to a final concentration of 0.5%, equal to the experimental wells. Invertebrate or fungal extracts were diluted in ICM and cells were treated with a final concentration of 150 μg/mL invertebrate extract and 125 μg/mL fungal extract. Each extract was tested in triplicate on 3 different hMSCs donors. Plates were covered with Breathe Easy sealing membrane and incubated 14 days at 37 °C, 5% CO_2_, 2% O_2_. Cells were supplied of 40 μL fresh medium at days 2, 4, 7 and 11. Differentiation was measured as the sulphated-glycosaminoglycans (*s*-GAG) secreted by the cells during differentiation. Fifty microliter of cell growth medium was discarded from all wells; 10 μL of 2 mg/mL papain (*Papaya latex*, Sigma) was added to each well to digest the extracellular matrix; plates were covered with sealing mats (ThermoFisher, Waltham, MD, USA) and incubated for 5 h at 60 °C. Quantification of *s*-GAG was performed using a dimethyl methylene blue staining (DMMB) assay. Briefly, 25 μL of the digested sample was added to 175 μL DMMB and absorbance immediately read at 595 nm using the Victor X5 plate reader. Three human MSCs donors were tested to evaluate retention of bioactivity. 

### 4.8. High-Throughput Anti-Inflammatory Assay

Immortalized macrophages (THP1) were stimulated to an inflammatory state by exposure to 100 ng/m bacterial lipopolysaccharide (LPS) in the growth medium. Cells (12.5 × 10^3^) were seeded in 384-well plates using the Janus Automated Workstation (PerkinElmer). Cells were then treated with 4 fungal extracts dilution (125, 62.5, 31.2, 15.6 µg/mL) prepared in cell growth medium and plates were incubated for 4 hours at 37 °C, 5% CO_2_. Negative control cells were treated with LPS alone; positive control cells were not exposed to LPS and in both cases cells were added of DMSO to a final concentration of 0.5%, equal to the experimental wells. Inflammation after incubation was quantified through measurement of TNFα retrieved in the conditioned medium using an automated immunoassay (TNFα DuoSet ELISA, R&D Systems, Minneapolis, MN, USA). Plates were spun for 5 minutes at 250 g, 40 μL conditioned medium was aseptically moved to a new plate, diluted five-fold in sterile PBS and 20 μL were dispensed to a pre-coated ELISA plate. Further steps were performed according to the manufacturer’s instructions using 1/5 of reagent volumes than what was indicated in the original protocol. At the completion of the immunoassay procedure, absorbance was immediately read at 540 nm and 531 nm using the Viktor X5 plate reader (PerkinElmer).

Anti-inflammatory hit re-screening was performed by quantification of TNFα and IL1β using appropriate ELISA kits and following the same modified procedure described. Untreated cells at increasing concentrations (12.5 × 10^2^, 2.5 × 10^2^, 5 × 10^3^, 1 × 10^4^, 2 × 10^4^, 2.5 × 10^4^) were included in the screening plates as a comparative standard to quantify the cell number in experimental wells after treatment. THP1 cells number was measured using AlamarBlue (ThermoFisher): after the conditioned medium used for the immunoassay was removed, 10% dye was aseptically added to the cells and incubated for 8 h at 37 °C, 5% CO_2_. An additional negative control was included in the initial screening plate containing 10% DMSO as cytotoxic agent. Fluorescence indicating AlamarBlue metabolism by active cells was measured at 530 nm excitation and 590 nm emission using Viktor X5 plate reader (PerkinElmer).

### 4.9. LC-MS Analysis of Extracts

Extracts were dissolved in acetonitrile or aqueous acetonitrile (1:1) for lipophilic and polar extracts respectively, to a final concentration of 0.5 mg/mL. The samples were filtered through a 0.2 µm filter prior to analysis. A 5 µL aliquot was injected onto a diphenol column (Fortis, 150 × 2.1 mm, 1.7 µm) and eluted using a mixture of acetonitrile/water (1% formic acid) at a gradient of 5% MeCN to 100% over 12 min, with the final solvent concentration held for 3 min. Solvent was delivered at a constant flow rate of 400 µL/min. HRESIMS and HRESIMSMS data were obtained using an Agilent 6540 Q-Tof mass spectrometer. Untargeted MSMS data was obtained using a collision energy of 10, 20 and 40 eV in positive mode. These data were then imported into the GNPS website for further analysis [41]. 

## 5. Conclusions

This study represents the first large screening of deep-sea extracts for their bioactivity toward stem cell differentiation and immunomodulation relevant for chronic disease drug discovery. Our platform allowed time-effective bioactivity screening following an experimental procedure based on advanced cellular assays. Screening of large libraries of crude extracts was motivated by the need to save rare and difficult-to-access samples for targeted chemical extractions, as well as to direct chemistry efforts to prioritized samples. Our results highlighted sea pens as the most promising invertebrates able to influence stem cell differentiation, especially the genera *Kophobelemnon* and *Anthoptilum*. Harvested samples of these genera and other sea pens are currently being extracted at larger scale aiming at pure compound isolation to generate a library of pure molecules for further testing. High-throughput screening of crude fungal extracts made possible simultaneous testing of multiple fungal species stimulated with various epigenetic modifiers, enabling rapid detection of the conditions leading to the production of bioactive molecules for our targets. Eight fungal strains are currently being cultured at higher scale for pure compounds isolation and further testing aiming at bioactive molecules identification. In both cases, bioprospection was used to identify and validate promising organisms from a large collection to efficiently direct the next steps in the biodiscovery pipeline. Deep-sea coral gardens are highly biogenic habitats formed by benthic invertebrates that develop over hundreds of years, hosting complex microbial communities [45]. Such habitats are critical for deep-sea ecosystem functioning and are endangered by overexploitation activities, such as fishing and mining, as well as ocean acidification [46,47]. Their extinction would severely impact ocean health and our chances to discover novel therapeutic compounds from largely understudied natural products sources.

## Figures and Tables

**Table 1:** Invertebrate extracts bioactivity.

Organism	Polarity	Bioactivity				
		Pro-Osteogenic	Pro-Chondrogenic	Proliferative	Anti-prolif.	Cytotoxic
*Acanella sp.1*	+		x			
*Anthoptilum* sp.	+		x			
*Anthothela sp.*	−				x	
*Anthothela* sp.1	−					x
*Anthothela* sp.2	+				x	
*Anthothela* sp.3	−				x	
*Balticina finmarchica*	−	x				
*Candidella imbricata*	+			x		
*Characella pachastrell.*	−			x		
*Chrysogorgia* sp.1	−				x	
*Chrysogorgia* sp.2	−				x	
*Chrysogorgia* sp.3	+				x	
*Chrysogorgia* sp.4	+					x
*Clavularia* sp.	−				x	
Demospongiae 1	−	x				
Demospongiae 2	−			x		
Hexactinellida	−	x				
*Keratoisinae* sp.1	−				x	
*Keratoisinae sp.2*	+		x			
*Keratoisinae sp.3*	+	x				
*Kophobelemnon sp.1*	−	x				
*Kophobelemnon sp.2*	−	x	x			
*Leiopathes* sp.	−	x				
*Muriceides* sp.	+	x				
*Polymastia arctica*	−			x		
*Radicipes* sp.1	−				x	
*Radicipes* sp.2	−			x		
*Radicipes* sp.2	+			x		
*Stichopathes* sp.1	−					x
*Stichopathes* sp.1	+				x	
*Swiftia pallida*	+		x			
Unidentif. sponge	−	x				
Zoanthid 1	−				x	
Zoanthid 1	+				x	
Zoanthid 2	−					x
Zoanthid 2	+				x	
Zoanthid 3	−				x	x
Zoanthid 4	−	x			x	
Zoanthid 5	+					x
Zoanthid 6	+			x		
Zoanthid 7	−	x	x			
Zoanthid 7	+			x		

In table are reported extracts showing proliferative or anti-proliferative activity (Anti-prolif.) compared to initial number of seeded cells (10^4^) as well as cytotoxic extracts inducing significant decrease of initially seeded cells. Pro-differentiation extracts reported are those validated on multiple hMSC donors.

## Supplementary Materials

The following are available online at https://www.mdpi.com/article/10.3390/md19070390/s1, Figure S1: High Throughput Screening deep-sea invertebrate extracts toward hMSCs. Figure S2: High- Throughput Screening deep-sea fungal extracts toward hMSCs. Figure S3: anti-inflammatory high- throughput screening of fungal extract library. Figure S4: anti-inflammatory high-throughput assay scale up to 384-well plate.
