# Supplementary material for: Deep-Sea Coral Garden Invertebrates and Their Associated Fungi Are Genetic Resources for Chronic Disease Drug Discovery
Source: Mar Drugs. 2021 Jul 13;19(7):390. doi: 10.3390/md19070390 (PMC8303266; doi:10.3390/md19070390)
Supplement: Supplementary file 1 [file marinedrugs-19-00390-s001.zip › marinedrugs-1265222-SI.pdf]

Supplementary Materials

Deep-sea coral garden invertebrates and their associated fungi are genetic resources for chronic disease drug discovery

Pietro Marchese[1,4], Ryan Young[2], Enda O'Connell[3], Sam Afoullouss[2], Bill J. Baker[4], A. Louise Allcock[2], Frank Barry[1], J. Mary Murphy[1]

[1] Regenerative Medicine Institute, School of Medicine, National University of Ireland Galway, H91TK33 Galway, Ireland; p.marchese1@nuigalway.ie;
[2] Martin Ryan Institute and School of Natural Sciences, National University of Ireland Galway, University Road, Galway, H91 TK33 Ireland
[3] Genomics and Screening Core, National University of Ireland Galway, H91TK33 Galway, Ireland
[4] Department of Chemistry, University of South Florida, Tampa, FL 33620, USA

* Correspondence: p.marchese1@nuigalway.ie

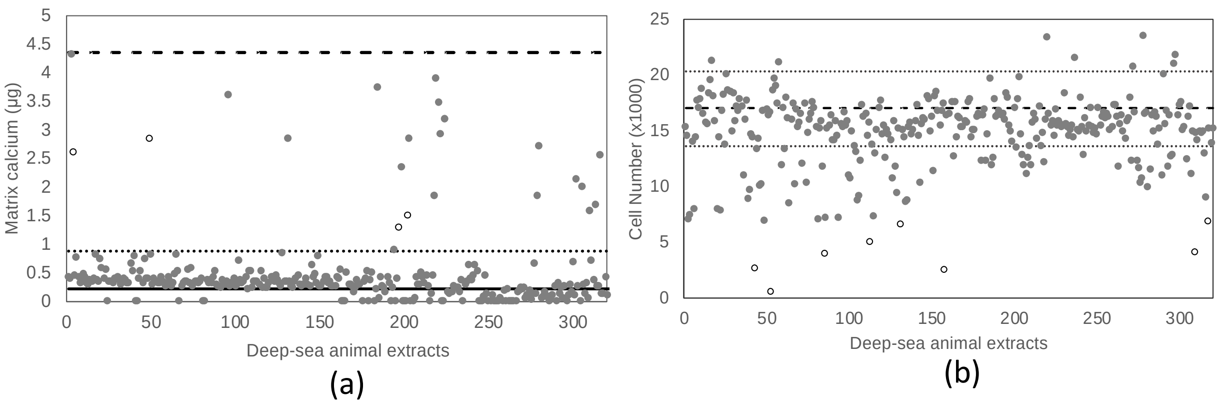

Figure S1. High Throughput Screening of deep-sea invertebrate extracts toward hMSCs. (a) Calcium-associated extracellular matrix mineralization after treatment with the deep-sea animal library. Extracts were dissolved in IOM medium lacking dexamethasone, cells were treated with a final extract concentration of 150 µg/mL and plates were incubated at 37 °C, 5% CO_2 for 10 days. Solid line indicates the negative control value (cells without dexamethasone), dotted line the screening threshold (Ctr+ average + 3*standard deviation) and the dashed line the positive control value (cells treated with OM medium containing 100 nM dexamethasone). White dots are compounds excluded from further screening because of antiproliferative activity. (b) Cell number recorded after treatment with deep-sea animal extracts as described above. Live cells were counted in each well after Hoechst 33342 nuclear staining using the Operatta high content imaging system. Dashed line indicates the positive control cell number, dotted lines are the higher and lower level of screening thresholds (Ctr+ average +/- 3*standard deviation). In white are highlighted extracts with cytotoxic activity as influencing a number of cells after treatment lower than the initially seeded number of cells (10^4).

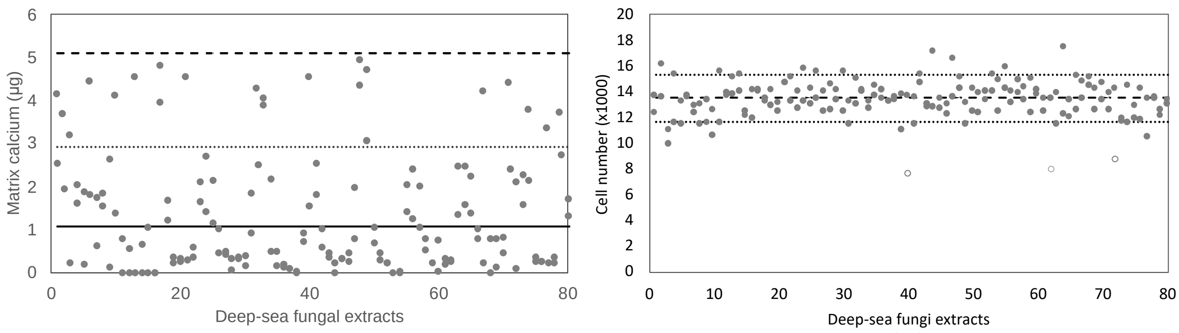

(a) (b)

Figure S2. High Throughput Screening deep-sea fungal extracts toward hMSCs. (a) Calcium extracellular matrix mineralization after treatment with the deep-sea fungal library. Extracts were dissolved in IOM medium lacking dexamethasone and cells treated with a final extract concentration of 125 µg/mL and plates were incubated at 37 °C, 5% CO_2 for 10 days. Solid line indicates the negative control value (cells without dexamethasone), dotted line the screening threshold (Ctr+ average + 3*standard deviation) and the dashed line the positive control value (cells treated with OM medium containing 100 nM dexamethasone). White dots are compounds excluded from further screening as showing antiproliferative activity. (b) Cell numbers recorded after treatment with deep-sea fungal extracts, as described above. Live cells were counted in each well after Hoechst 33342 nuclear staining using the Operetta high content imaging system. Dashed line indicates the positive control cell number and dotted lines are the higher and lower level of screening thresholds (Ctr+ average +/- 3*standard deviation). In white are highlighted cytotoxic extracts that that after treatment induced lower cell numbers than that initially seeded (10^4).

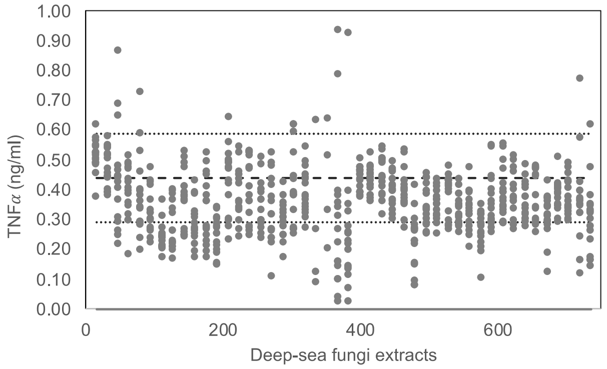

Figure S3: anti-inflammatory high throughput screening of the fungal extract library. One hundred-sixty fungal extracts were tested on LPS activated THP1 macrophages to evaluate their potential to decrease the cellular inflammation state. Negative control cells (dashed line) were treated with 100 ng/mL LPS, while positive control cells were cultured in growing medium only. Positive hits were selected according to the screening threshold (Ctr- average - 3*standard deviation). Selected hits showed bioactivity lower than the threshold for at least two of the four concentrations tested and were non cytotoxic.

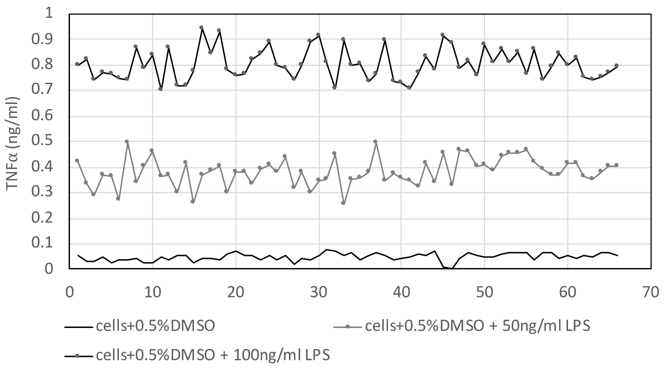

Figure S4: **Anti-inflammatory high throughput assay scaled to a 384-well plate**. Twelve thousand THP1 cells were seeded in a 384-well plate and treated with either growing medium, LPS to a final concentration of 50 ng/mL or 100 ng/mL. The level of TNFα present in the conditioned medium was quantified through an automated ELISA performed in a 384-well plate. Cytokine level comparison between untreated cells and the two concentration of LPS was used to calculate the Z′ factor: an optimal score (>0.7) was obtained using LPS at the higher concentration, and therefore 100 ng/mL was used to treat cells in the HT assay.
